# Effect of Dielectric and Liquid on Plasma Sterilization Using Dielectric Barrier Discharge Plasma

**DOI:** 10.1371/journal.pone.0070840

**Published:** 2013-08-07

**Authors:** Navya Mastanaiah, Judith A. Johnson, Subrata Roy

**Affiliations:** 1 Applied Physics Research Group (APRG), Department of Mechanical and Aerospace Engineering, University of Florida, Gainesville, Florida, United States of America; 2 Department of Pathology, Immunology and Laboratory Medicine, College of Medicine and Emerging Pathogens Institute, University of Florida, Gainesville, Florida, United States of America; University Paul Sabatier, France

## Abstract

Plasma sterilization offers a faster, less toxic and versatile alternative to conventional sterilization methods. Using a relatively small, low temperature, atmospheric, dielectric barrier discharge surface plasma generator, we achieved ≥6 log reduction in concentration of vegetative bacterial and yeast cells within 4 minutes and ≥6 log reduction of *Geobacillus stearothermophilus spores* within 20 minutes. Plasma sterilization is influenced by a wide variety of factors. Two factors studied in this particular paper are the effect of using different dielectric substrates and the significance of the amount of liquid on the dielectric surface. Of the two dielectric substrates tested (FR4 and semi-ceramic (SC)), it is noted that the FR4 is more efficient in terms of time taken for complete inactivation. FR4 is more efficient at generating plasma as shown by the intensity of spectral peaks, amount of ozone generated, the power used and the speed of killing vegetative cells. The surface temperature during plasma generation is also higher in the case of FR4. An inoculated FR4 or SC device produces less ozone than the respective clean devices. Temperature studies show that the surface temperatures reached during plasma generation are in the range of 30°C–66°C (for FR4) and 20°C–49°C (for SC). Surface temperatures during plasma generation of inoculated devices are lower than the corresponding temperatures of clean devices. pH studies indicate a slight reduction in pH value due to plasma generation, which implies that while temperature and acidification may play a minor role in DBD plasma sterilization, the presence of the liquid on the dielectric surface hampers sterilization and as the liquid evaporates, sterilization improves.

## Introduction

Plasma makes up the majority of the universe. Natural and fabricated plasmas occur over a wide range of pressures, temperatures and electron number densities. Fabricated plasmas are ionized gases, made up of ions, electrons and neutrals. These are commonly categorized based on either temperature or electron number density. In this paper, we are working with low temperature plasmas generated from room air.

**Figure 1:**
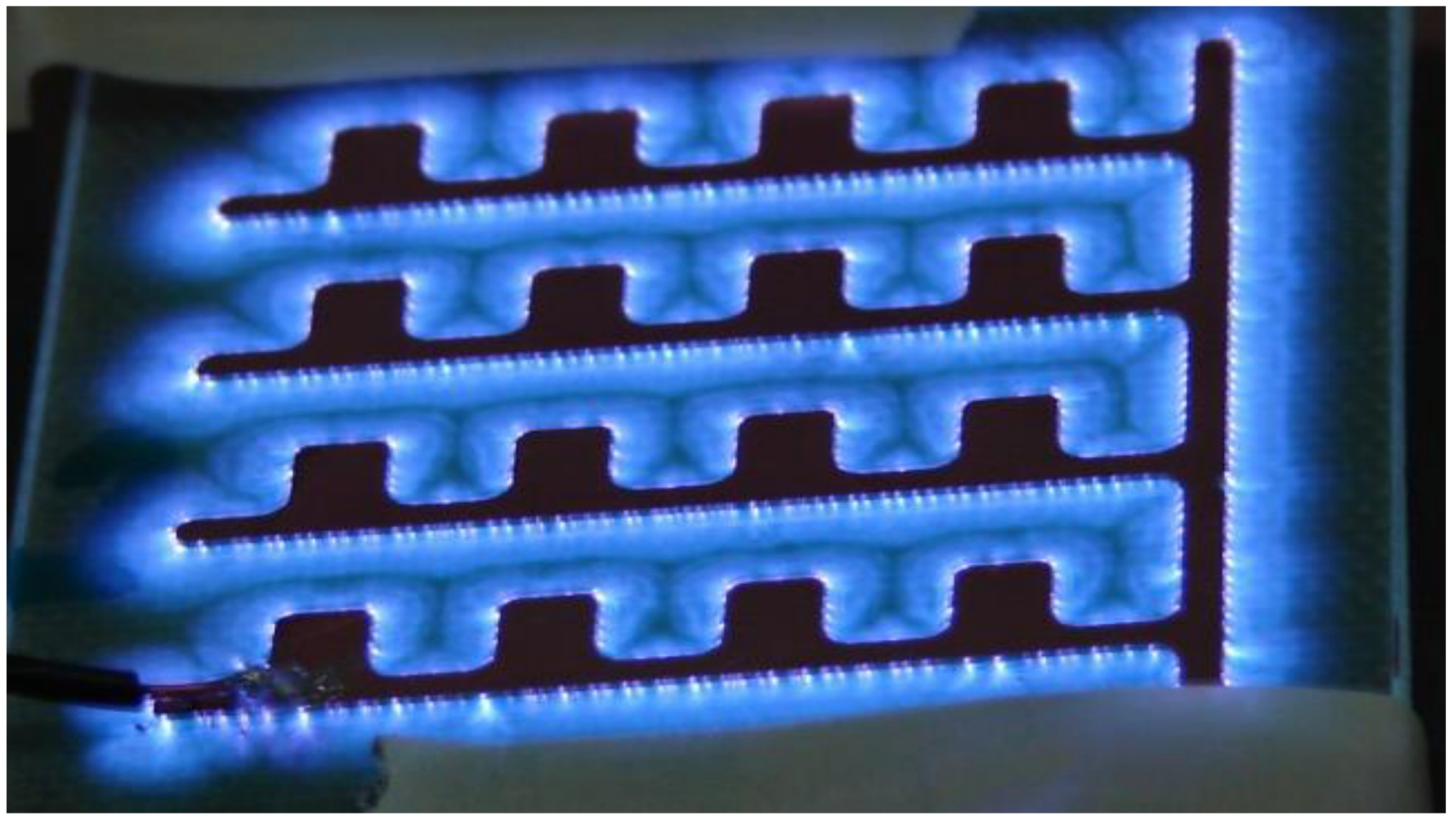
Plasma device (sawtooth electrode) used for earlier experiments in this paper.

Depending on the applied voltage and discharge current, different types of plasma discharges can be obtained [1]. A corona discharge occurs in regions of high electric field near sharp points in gases prior to electrical breakdown. The transition from the Townsend and corona discharge regime to the sub-normal and normal glow discharge regime is accompanied by a decrease in voltage and increase in current. The ‘glow discharge’ owes its name to the luminous glow seen during plasma generation as seen in Figure 1 below. For the purpose of this paper, this device is denoted as the *‘sawtooth electrode’.* The dielectric barrier discharge (DBD) surface plasma, which is the type of plasma discussed in this paper, occurs in the transition between corona and normal glow discharge.

**Figure 2:**
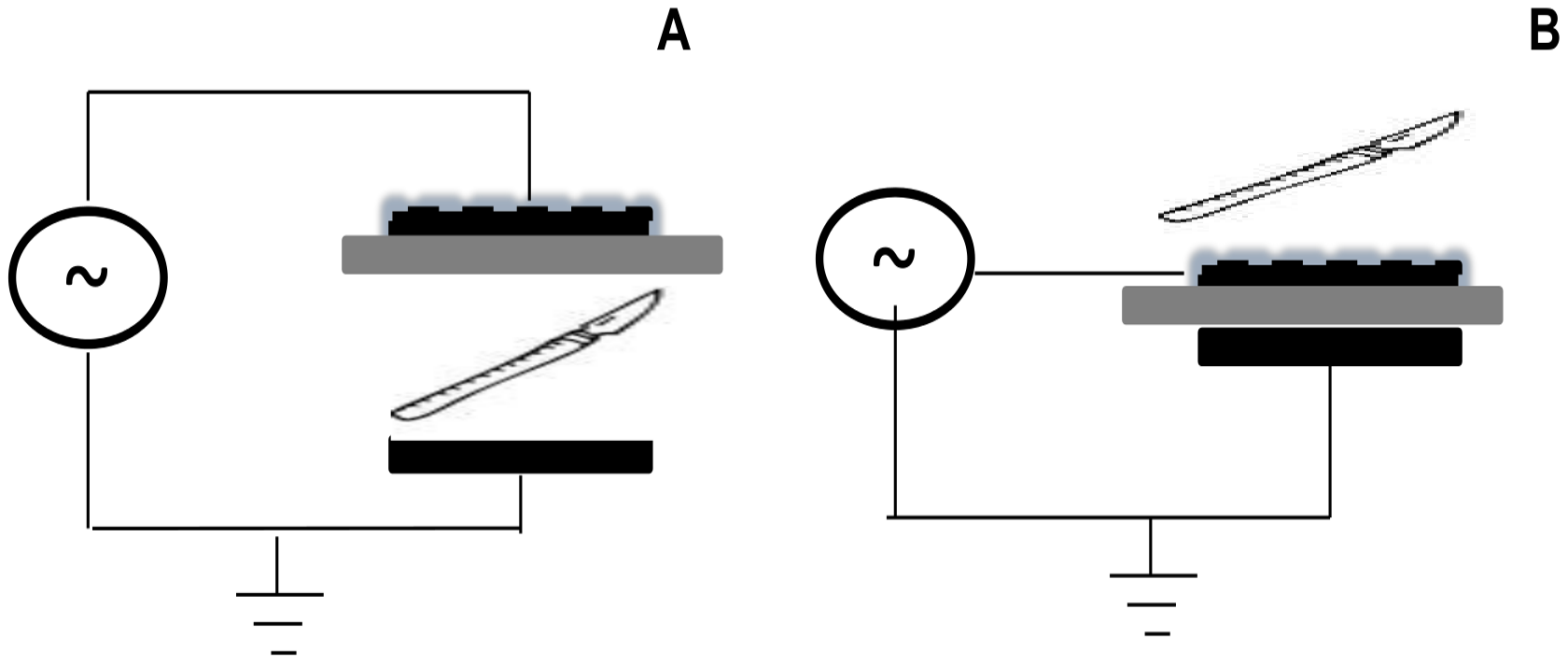
Schematic showing the difference between (A) Volume Plasma Configuration (B) Surface Plasma Configuration. In both (A) and (B), the powered electrode is the dashed, black surface on top. The grounded electrode is the solid, black surface on the bottom. The grey surface in between is the dielectric barrier. In (A), a small scalpel that needs to be sterilized would be placed in the discharge gap between the dielectric barrier and grounded electrode since this is where plasma is generated. In (B), the same small scalpel would be placed on top of the dashed black surface, since this is where the plasma would be generated.

DBD plasmas are a special class of plasmas that operate at pressures of 0.1–10 atm. They are effective ozonizers and are used in a number of additional applications such as surface modification, plasma chemical vapor deposition and most popularly, in large plasma display panels used in television [2]. In its simplest configuration, DBD is the gas-discharge between two electrodes, separated by one or more dielectric layers. Gap between electrodes is typically of the order of millimeters. A broad range of voltages (1–100 kV) and frequencies (50 Hz- 1 MHz) are required to sustain such a discharge. The presence of the dielectric barrier inhibits the transition from glow to arc, thus ensuring stable, non-thermal plasma. DBD plasmas can be classified into two configurations, as shown below in Figure 2.

The volume plasma configuration, shown in Figure 2 (A), consists of two electrodes separated in between by one or more dielectric layers and a discharge gap. Plasma is generated within this discharge gap. Much of the work with plasma sterilization has focused on this type of setup that sterilizes items within a chamber and there has been difficulty in attaining sterilization with air plasmas using this configuration [3]. The surface plasma configuration, shown in Figure 2 (B), differs from the volume plasma configuration, in that there is no discharge gap. Plasma is generated on the surface of the device. This paper focuses on surface plasma sterilization for which the discharge configuration consists of a dielectric layer, whose either side is embedded with electrodes such that plasma is generated atop a surface of the dielectric layer. The plasma is concentrated in a thin layer near the surface that sterilizes the surface containing the electrode as well as nearby surfaces. If the electrode is shaped to allow close proximity, this concentration may aide in sterilization.

Sterilization refers to any process that results in the complete elimination or destruction of all living microorganisms. Conventional methods of sterilization such as autoclaving, dry heat, ethylene oxide (EtO) fumigation and γ-irradiation, while established as effective methods, do have their disadvantages, especially damage to heat sensitive polymers, long processing times and/or the need for expensive and potentially dangerous equipment. The ideal sterilant as defined by Moisan et.al. [3] should provide (a) short sterilization (b) low processing temperatures (c) versatility of operation and (d) harmless operation for patients, operators and materials. Plasma sterilization provides advantages in all these criteria. Very short times have been reported by literature [4]–[5]. DBD plasma operates almost at room temperature. Plasma generated from ambient air produces a variety of reactive species such as oxygen and nitrogen ions as well as UV photons. Since most of these chemical species disappear milliseconds after the discharge is switched off, they do not leave any toxic residue. However, DBD devices are also known ozone generators, which contribute to killing, and any sterilization setup using these devices must be equipped with measures to control the excessive amount of ozone produced.

The origins of plasma sterilization can be traced back to a patent filed by Menashi [6] in 1968. Research in plasma sterilization can be traced along two parallel paths: 1) Determination of the optimum set of parameters for fast, safe plasma sterilization 2) Understanding the underlying mechanism of plasma sterilization. Early research in plasma sterilization was aimed at the former. Hury et.al. [7] conducted a parametric study wherein they tested the destruction efficiency of a 2.45 GHz plasma, generated in a cylindrical reactor using oxygen as the discharge gas, on *Bacillus subtilis* spores. Their studies confirmed previous assertions that oxygen plasmas achieved more killing than argon plasmas, with H_2_O_2_ and CO_2_ plasmas achieving high destruction efficiencies. Lerouge et.al. [8] conducted studies using a large volume microwave plasma reactor, wherein different gas compositions were compared in terms of spore destruction efficiencies. They found that O_2_/CF_4_ plasma achieved most effective sterilization, due to the combined etching action of both oxygen and fluoride atoms. Moreau et.al. [9] used the flowing afterglow of a 2.45 GHz microwave plasma to inactivate *B. subtilis* spores within 40 minutes.

Most of the papers cited above fall in the low pressure regime. A lot of recent work has been published in the atmospheric pressure regime. This recent body of research ranges in scope from the exploration of new plasma sources for sterilization to usage of diagnostic and microbiological tools to investigate the mechanism of plasma sterilization. Ying et.al. [10] compared yeast inactivation in helium(He), Air and nitrogen (N_2_) DBD (volume) plasma at atmospheric pressure. Working at a frequency of 0–20 kHz and an input voltage of 40 kV p-p for a treatment time of 5 minutes, they reported a 5-log reduction with N_2_, 6-log reduction with air and 7 log reduction with He. Sladek et.al. [11] reported atmospheric plasma interaction with *S. mutans* biofilms and concluded that a single plasma treatment for 1 minute on biofilms cultured without sucrose caused no re-growth within the observation period. Kalghatgi et.al. [12] took a more fundamental route in assessing the damge due to plasma exposure. They concluded that the effect of plasma ranges from increasing cell proliferation to inducing apoptosis (programmed cell death). Joshi et.al. [13] used anti-oxidants (compounds that protect bacteria from oxidative stress) to prove that when these agents were used to scavenge the reactive oxygen species produced during plasma generation, membrane lipid peroxidation and oxidative DNA damage was significantly inhibited, proving that the ROS causing oxidative DNA damage is a major mechanism involved in DBD plasma sterilization.

In spite of many years of study, plasma sterilization has still not become widely used. Optimization of plasma killing has been difficult due to the complexity of plasma and limited understanding of how it interacts with microbes. Moisan et.al. [3] wrote about the uncertainty concerning the role of UV in the process of plasma sterilization. While earlier experiments [14], mostly conducted in the low-pressure regime, believed that UV (especially in the VUV range (<200 nm)) was a primary factor in sterilization, later experiments conducted at higher pressure suggested that UV radiation was of less importance. Laroussi et.al. [15] used a DBD setup in the volume plasma configuration to record the UV spectrum of air plasma in which they noted there was no significant UV emission below wavelengths of 285 nm. Similarly, Dobrynin et.al. [16] reported experiments wherein they used a quartz filter (transparent to UV photons of >200 nm) during plasma treatment of bacteria They noted from these experiments that there was no visible effect on bacteria by UV/VUV radiation. However, they end with the note that the role of VUV should not be discounted completely.

Dobrynin et.al. also explored the plasma dosage required for bacterial inactivation in cases with and without water. Their results showed that the plasma dosage required for complete bacterial inactivation in cases with water was lower than that required for cases without water. They also concluded from other experiments in the same paper that the presence of water and direct plasma treatment were both required to achieve fast inactivation and this inactivation was highly dependent on the amount of water. Other approaches in understanding plasma sterilization have also included using various protein-detection assays to detect the leakage of a particular protein that might indicate the rupture of the cell wall [17]. Numerical models for plasma sterilization have also been proposed taking into account sterilization times and reaction constants from existing empirical data [5], [18].

This paper describes the implementation of a high-frequency DBD plasma source (operating at 14 kHz and low input power) to sterilize vegetative microbes and spores on a surface with applied electrodes. We compare two different dielectric substrates: FR4 (which is commonly used in manufacturing printed circuit boards and has a mean dielectric constant (ε) of 4.15) and a semi-ceramic laminate (RO3003®, which has a dielectric constant of 3.00±0.04). Plasma is characterized by the spectral signature and ozone levels produced. Further, we evaluate the effect of the liquid portion of the test culture on efficacy of sterilization. These experiments begin the process of optimizing plasma sterilization.

## Materials and Methods

### Experimental Setup

**Figure 3:**
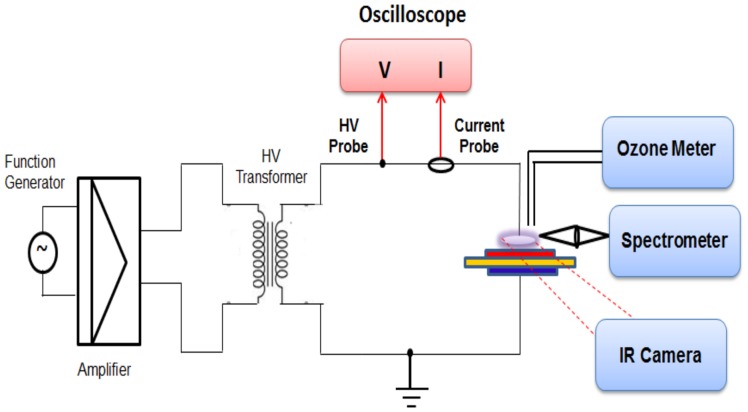
Schematic of the Experimental Setup used.

Figure 3 above shows the schematic of the experimental setup used in plasma generation and testing. A function generator (Agilent ® 33120A) is used to generate a sinusoidal RF signal of frequency 14 kHz. The power of this signal is then amplified using an amplifier (model Crown CDi4000). This amplified signal is then passed through a step-up transformer. The input power from the transformer is fed to the powered electrode (shown in red) of the device via a metal connector. This electrode configuration can also be flipped without affecting sterilization effectiveness, i.e. the red electrode can be grounded and the blue one powered. This allows one to design devices with a reduced risk of electrical shock due to touching of the powered electrode. The powered and grounded electrodes are separated by a sheet of dielectric material, about 1.6 mm thick. In this paper, two types of dielectric material are considered and compared: FR4 (Flame Retardant 4) and Rogers®3003 semi-ceramic (SC) dielectric with ε = 3.00±0.04. FR4, which is commonly used for making printed circuit boards, has a dielectric constant (ε) of 3.8–4.5 (mean 4.15). Both dielectric sheets are overlaid with a copper layer and are etched out, according to the requisite electrode pattern.

To compare characteristics of the plasma generated using these two dielectric materials, input voltage and current are measured using an Agilent ® DSO1004 Oscilloscope and a current probe. The final input signal into the plasma device has a power ∼7–10 W and an input voltage of 12 kV peak-to-peak (p-p). The other electrode (shown in blue) of the device is connected to an electrically grounded bench, atop which the device sits. Before any experiment, the experimental bench was swabbed with 70% proof ethyl or isopropyl alcohol and allowed to completely dry so that a clean testing environment was maintained. Following sterilization trials, plasma devices were removed from the bench and placed in sample bags for microbiological testing.

The plasma device consists of a dielectric square that measures 3.5×3.5 cm^2^. This dielectric is embedded with the bottom and top electrodes (on either side). The bottom (grounded) electrode is a square sheet of metal, measuring 2.4×2.4 cm^2^. The powered electrode has a *comb-like* design that has the same surface area as the grounded electrode. Earlier configurations of these plasma devices also incorporated a *sawtooth* design for the powered electrode.

**Figure 4:**
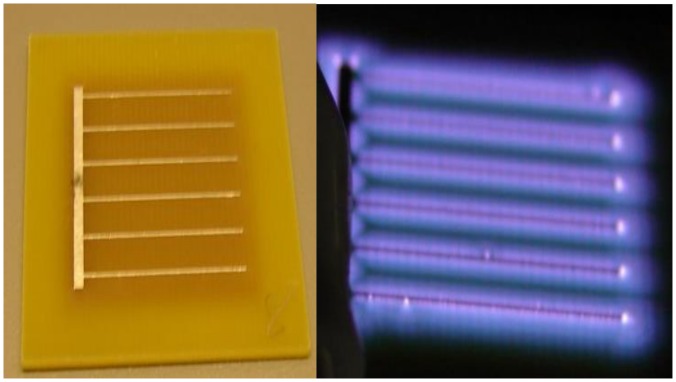
The plasma device used for sterilization experiments in this paper (A) un-powered (B) powered (generating plasma).

Two electrode configurations are used in this paper. Figure 4(A) above shows the type of device that has been used for most of the experiments in this paper. For the purpose of this paper, this device is denoted as the *‘comb electrode’*. Figure 4(B) shows the same device; powered and producing glow discharge. Note that the plasma seen in this figure covers the entire electrode surface area, contrary to the *‘sawtooth electrode’* show in Figure 1. In the *sawtooth electrode*, parts of the electrode surface area are enveloped by the plasma glow and parts of it (the electrodes themselves) are not. The significance of the plasma glow enveloping the entire electrode surface area will be described later while discussing the sterilization curves.

The spectroscopic signature of the generated DBD plasma is determined using the Ocean Optics® USB 2000+ spectrometer. This spectrometer has a detector range of 200–1100 nm, an optical resolution of ∼0.3–10 nm (FWHM), a dynamic range of 1300∶1 for a single scan and is fitted with a custom-made grating designed to be sensitive to wavelengths between 200–650 nm. An uncoated UV Fused Silica Plano-Convex Lens (Ф2′′, f = 75 mm) is used to collect and focus the incident plasma glow from the plasma device, which is then detected by the spectrometer via a fiber-optic probe. Baseline data for each device was collected with the device powered for 2 minutes while its spectral signature was recorded every 10 s. Readings were also taken during sterilization experiments.

A 2B Tech® Ozone meter is used to measure the ozone levels at fixed time intervals within a closed chamber. This ozone meter operates on the principle that the maximum absorption of ozone takes place at 254 nm. Air is sampled every 10s and the sampled ozone levels are saved to a computer via a LabView ® Interface. Surface temperature of the dielectric substrate during plasma generation is measured using an infrared camera (FLIR A320®). The A320 operates at a spectral range of 7.5–13 µm and has a pixel resolution of 320×240 pixels. The distance between the plasma device and infrared camera, ambient temperature and humidity and the emissivity of the FR4, SC dielectric are measured to be 0.2667±0.0127 m, 24.4±2.3°C, 59±3% RH and 0.9097±0.03, 0.929±0.03 respectively.

### Microbiological Testing

Cultures were maintained frozen at −80°C in broth with 25% glycerol and inoculated onto fresh plates weekly. *Saccharomyces cerevisiae* (Fleischmann’s Baker’s Yeast) was grown on Sabouroud’s (SAB) agar at 30°C overnight. A single colony was inoculated into SAB broth and incubated at 30°C with shaking. *Escherichia coli C600* was grown on Luria-Bertani (LB) agar or broth at 37°C. Purified *Geobacillus stearothermophilus* spores (SCM Biotech, Bozeman, MT) at 3.1×10^7^ spores/ml, in alcohol, were stored at −20°C and grown on trypticase soy (T-soy) agar or broth at 50°C. Before each experiment, the optical density (OD) of the microbial sample was measured using an Ultrospec 10 cell density meter (GE Healthcare Bio-Sciences Corp., Piscataway, NJ) to estimate the density of the culture. An OD of 1 corresponds to approximately 5×10^8^ colony forming units (CFU) for *E. coli* and 1×10^8^ CFU for *S. cerevisiae.* Cultures were diluted if needed to ensure that approximately 10^6^ CFU were inoculated on the device.

For each experiment, the plasma devices were inoculated with 20 µl of the bacterial sample (unless otherwise noted) spread uniformly over the entire surface area of the top electrode, using a sterile inoculating loop. A separate plasma device was used for each time-point. After each experiment, plasma devices were either autoclaved (for spore experiments) or disinfected with 70% ethyl alcohol and sealed in sterile bags. Once the experiment was completed, each tested device was deposited in a sterile bag with 5 ml of appropriate culture broth. The bag was sealed and agitated thoroughly using a Fisher Scientific ® Mini Vortexer Lab Mixer to detach any microorganisms clinging to the device. Serial dilutions were spread on appropriate plates that were then incubated at the required temperature for 24–48 hours and counted. Plate counts were also performed on the inoculum to determine the exact concentration of organisms and an inoculated device not exposed to plasma was processed to control for loss of viable counts due to drying or adherence to the device. Experiments were performed in triplicate unless otherwise noted.

## Results

### Sterilization Curves Using Several Vegetative Microbes and Spores as Test Pathogens

**Figure 5:**
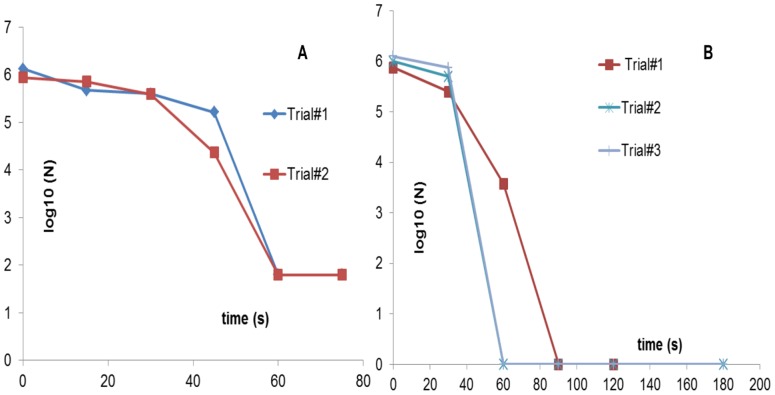
Sterilization plots obtained using *S. cerevisiae* (yeast) with (A) sawtooth electrode (B) comb-like electrode. Earlier sterilization trials were conducted using the sawtooth configuration. However, with extended usage, the sawtooth configuration began posing a problem, which is why a new comb-like electrode was designed and implemented.

In testing out our in-house DBD plasma sterilization setup, baker’s yeast (*S. cerevisiae)* was used for preliminary tests of killing of vegetative cells by plasma. The sterilization plots from these trials are shown below in Figure 5(A). These tests were conducted using an input frequency of 14 kHz and an input voltage of 12 kVp-p for plasma generation. The DBD devices used had the *sawtooth electrode* configuration, shown in Figure 1.

While these tests showed a 5 log reduction in 75s, subsequent tests started yielding inconsistent data. It was soon realized that the electrode configuration was at fault. As is seen in Figure 1, when the device is powered, the electrode itself is not covered by plasma (enveloped by the bluish glow) unlike the rest of the dielectric surface. This led to contaminated areas atop the electrode surface that did not seem to be completely sterilized by the generated plasma as was confirmed by placing the electrode, inoculated surface face down on a SAB agar plate following plasma exposure. Colonies only grew on the area covered by the electrode (data not shown). Hence, it was realized that thinner electrodes placed with an optimum gap in between led to uniform plasma coverage over a surface, thus helping uniform sterilization. This led to the second electrode configuration (*comb-like*) shown in Figure 4, where the generated plasma is seen enveloping the entire electrode and dielectric surface. Using the new electrode configuration, consistent sterilization times of 60s–90s were observed in the case of yeast (as shown above in Figure 5(B)).

**Figure 6:**
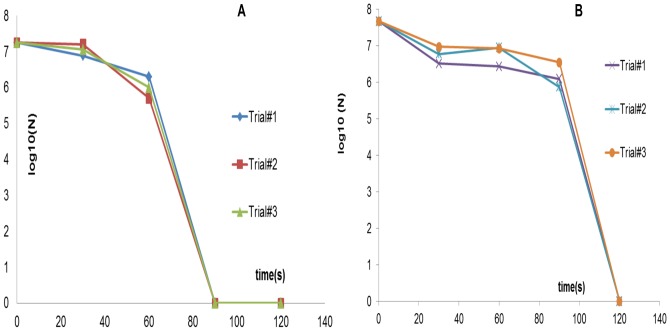
Sterilization plots obtained using *E. coli* as the test pathogen using (A) FR4 dielectric (B) semi-ceramic (SC) dielectric. The former achieves complete sterilization, starting from an initial concentration of 10^7^ cfu in 90s, while the latter achieves the same in 120s.

To test for killing of Gram-negative vegetative bacterial cells, *E. coli C600* was plasma treated for different time intervals and the sterilization plot is shown in Figure 6(A) below. 10^7^ cfu were killed within 90s using the FR4 dielectric plasma devices. The same sterilization trials using the semi-ceramic (SC) dielectric plasma devices resulted in 10^7^ cfu being killed within 120s. This result is also shown below in Figure 6(B). Both sterilization plots show a phase with little or no loss of viability followed by a rapid killing of the test sample.

**Figure 7:**
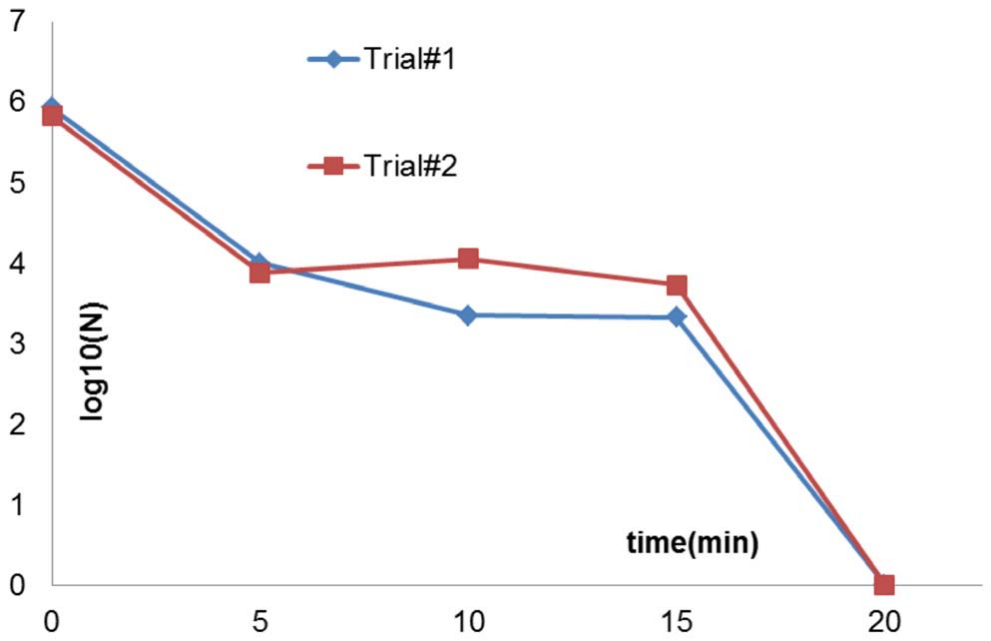
Sterilization plots using *G. stearothermophilus* as the test pathogen. FR4 dielectric was used for these tests. Complete sterilization, starting from an initial concentration of 10^6^ cfu was obtained in 20 min.

Spores are tough, dormant, non-reproductive organisms produced by some bacteria as a survival mechanism when threatened by harsh conditions. Sterilization, by definition, requires the ability to kill bacterial spores. Purified *G. stearothermophilus* spores were used as the spore challenge. In this case, the inoculation volume used was 40 µl. As shown in Figure 7 below, the sterilization curve for spores shows a triphasic pattern with a 2-log reduction in the first 5 minutes, a slow killing period, and complete inactivation (6-log reduction) within 20 minutes.

### Spectroscopic Studies

**Figure 8:**
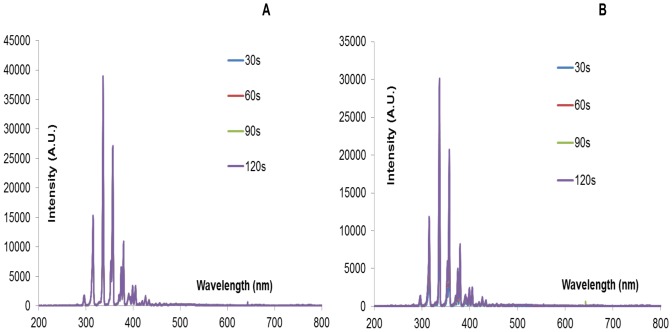
Spectral signature of (A) a clean FR4 device (B) an inoculated FR4 device i.e. a device on which 20 µl of *E. coli* was applied uniformly. Spectral signature was recorded every 10s. Devices were powered for a total of 2 minutes. Only spectra at 30s, 60s, 90s and 120s are shown. Y-axis lists emission intensity in arbitrary unit.

Spectral signatures of the devices were recorded before and during sterilization experiments. For the spectral signatures obtained in Figure 8 below, both clean and inoculated FR4 plasma devices were powered for a total of 2 minutes.

Intensity peaks observed at particular wavelengths can be compared to existing literature [19] in order to identify the molecular species responsible for the respective intensity peaks. Two dominant intensity peaks are observed at wavelengths 337.13 nm and 357.7 nm. Both correspond to the 2^nd^ positive system of N_2_ (C^3^Π_u_-B^3^Π_g_). No intensity peaks are noted at wavelengths characteristic of O_2_ or O_3_ molecules. Laroussi et.al. [15] noted a similar result, using DBD plasma in volume configuration. Since the spectral signature shows no noticeable wavelengths below 290 nm, it is unlikely that shortwave UV radiation (200–300 nm) plays a major role in surface DBD plasma sterilization.

**Figure 9:**
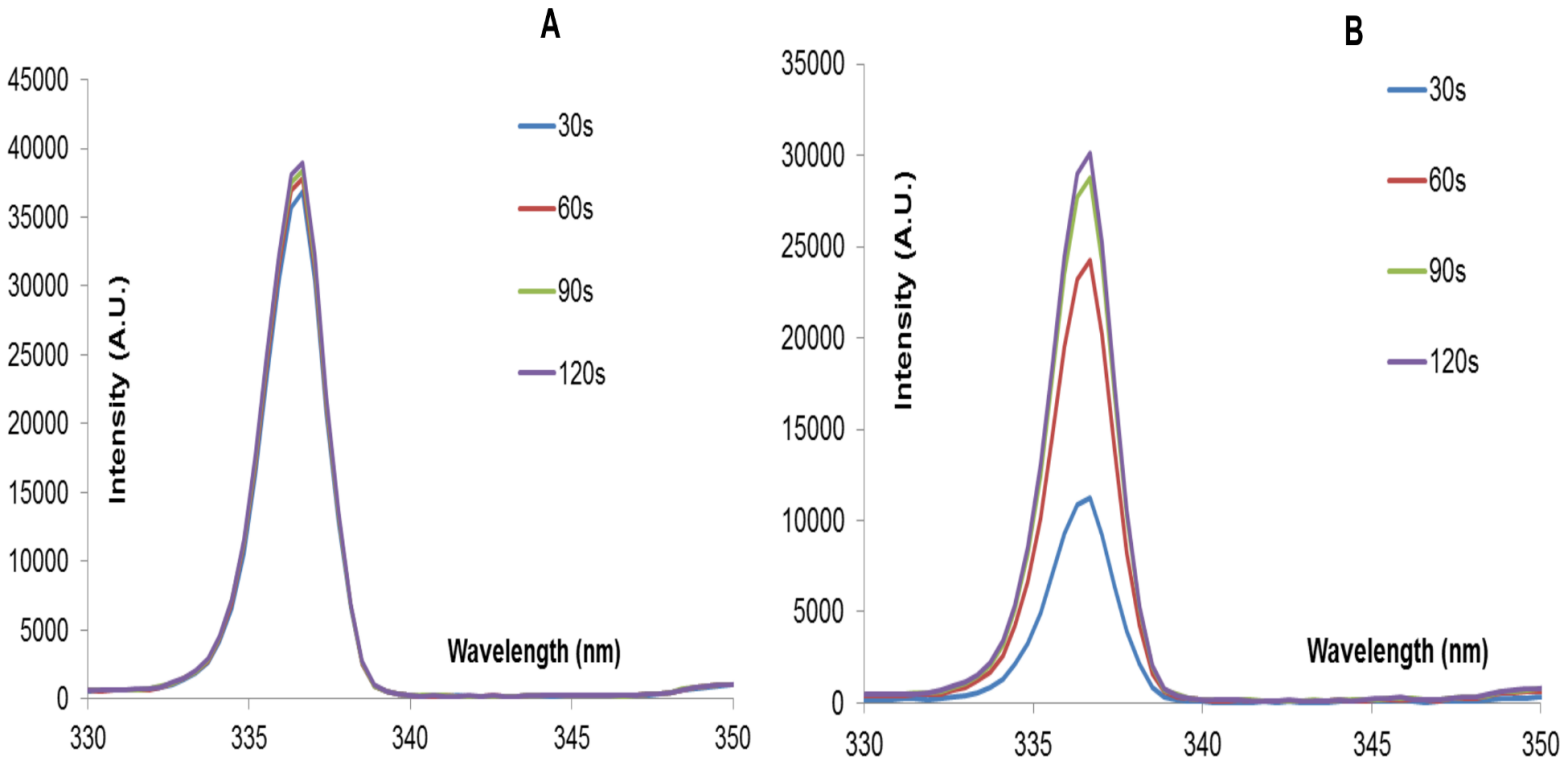
Expanded version of the spectral signature of (A) a clean FR4 device (B) an inoculated FR4 device. This is an expanded image of Figure 8, wherein, the intensity peak in the wavelength range 330–350 nm is depicted to highlight the intensity difference between the clean and inoculated case at different sampling times (30s, 60s, 90s, and 120s).

From Figure 8 above, it is seen that the peak intensities occur in the wavelength range 300–500 nm. Figure 9 below is an expanded version of Figure 8, focusing on the spectral signature in the range 330–350 nm.

In Figure 9(A) above, the spectral data sampled at 30s, 60s, 90s and 120s during the 2-minute interval is shown. All four plots show similar peaks i.e. Plasma generated using a clean FR4 device for a 2-minute time interval, shows similar intensity values at all times. However, it is evident in Figure 9(B) that the intensity value at 30s is less than that at 120s. As time progresses, it is also observed that the amount of liquid bacterial sample on the inoculated device decreases and intensity increases. This dependence of spectroscopic intensity on amount of liquid bacterial sample is discussed in detail later on.

### Ozone Studies

Ozone is an effective bactericidal agent and may play a role in the sterilization process. It is also a respiratory irritant that must be controlled to protect the device operator. Thus, it is important to understand how much ozone is produced and how fast it dissipates. With such a high amount of ozone produced, it is highly important that we understand factors such as the rate of production/dissipation of ozone, its dependence on different dielectrics as well as necessary precautions to be taken for safe operation of these devices.

**Figure 10:**
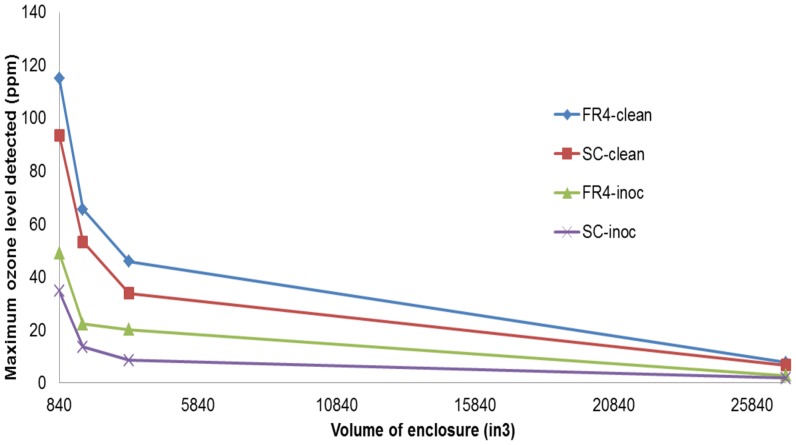
Variation of the maximum ozone levels w.r.t the volume of the acrylic enclosure. Four plots are shown. Each plot shows the maximum ozone level noted in each enclosure for the respective device. “FR4-clean” shows this plot for a clean FR4 device, generating plasma for 1 minute. “FR4-inoc” shows this plot for an inoculated FR4 device, generating plasma for 1 minute. “SC-clean” shows this plot for a clean semi-ceramic (SC) device, generating plasma for 1 minute. “SC-inoc” shows this plot for an inoculated semi-ceramic (SC) device generating plasma for 1 minute.

In order to do this, a 2B Tech ® Ozone meter was used to measure the ozone levels while the plasma devices (both clean and inoculated) were powered for 1 minute. The plasma device and ozone meter were set up in an acrylic enclosure to allow accurate measurement of the concentration of ozone. Enclosure sizes of varying volumes were tested in order to understand how the volume of the enclosure affected the concentrations of the emitted ozone and its subsequent diffusion and breakdown. The volume of the smallest enclosure was 840 in^3^. The ozone probe was placed 6.5″ above the chamber floor and ∼1″ away from the device. The ratio of the volumes of enclosures #2,3,4 w.r.t to the smallest enclosure (#1) was 2∶4∶32. Figure 10 below shows this dependence. The X-axis denotes volume of the enclosure (in^3^) while the Y-axis denotes ozone levels (ppm).

**Figure 11:**
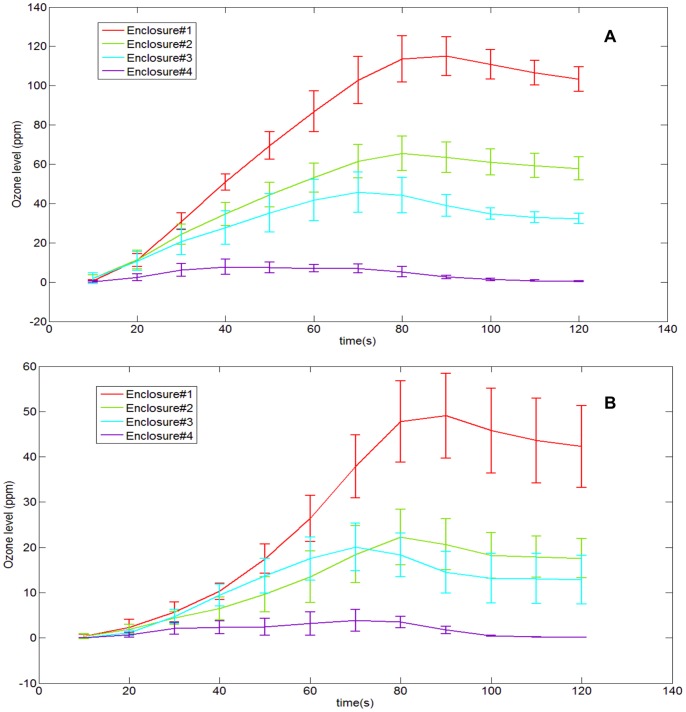
Depicting the trend of ozone production and dissipation for four different acrylic enclosures of different volumes using A) Clean FR4 devices (B) Inoculated FR4 devices. These devices were powered for 1 minute. Ozone data is sampled every 10s.

From Figure 10, the following observations are made. The clean FR4 device generates the maximum amount of ozone, followed by the clean SC device (∼28% less). Both the inoculated FR4 and SC device generate considerably lesser amounts of ozone than their clean counterparts (∼ 70% higher) do. Figure 11 given below depicts this difference in ozone production between a clean and inoculated device. For the sake of simplicity, this difference is shown only for the FR4 dielectric.

Two observations are made from Figure 11 (A) and (B) above. The first observation reinforces that made in Figure 10 i.e. as the volume of the enclosure decreases, the ozone concentration increases. This is because as the volume of the enclosure decreases, the concentration of ozone confined within the enclosure increases. Secondly, the large difference in ozone concentrations between a clean and an inoculated FR4 device is to be noted. In each enclosure, the maximum ozone level noted is about 20%–60% more in the clean case as compared to the inoculated case. This seems to be proportional to the amount of liquid present on the surface of the device. For an inoculated device, while plasma is generated, initially very low levels of ozone are produced. As the liquid evaporates, the amount of ozone produced increases. As with spectroscopic intensity, this dependence of produced ozone on the amount of liquid sample presents provides a significant insight into the plasma sterilization process and will be discussed later on.

### Power Measurements

**Figure 12:**
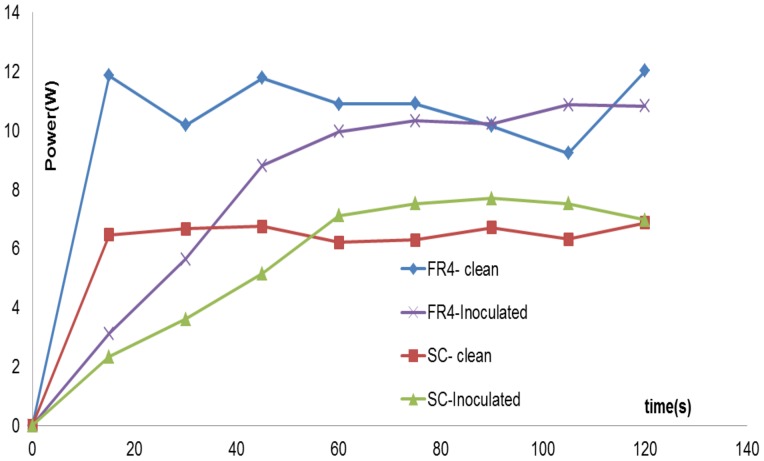
Variation of the input power over time for clean and inoculated FR4 and SC devices. Input power was sampled every 15s over a 2 minute interval for all four cases. Devices were powered during the entire 2-minute interval.

To better understand the mechanism of plasma sterilization, it was necessary to measure the input power being fed into both clean and inoculated FR4 and semi-ceramic (SC) devices. An experiment was performed in which an inoculated device was powered for 2 minutes. The input power to the device was measured every 15s, using the Agilent ® DSO1004 Oscilloscope and a current probe. Figure 12 below gives this plot of the power varying over time, both for the FR4 as well as semi-ceramic (SC) devices.

In Figure 12, the power varies between 10–12 W for a clean FR4 device. The average power measured over this 2 min interval is 9.67 W. Similarly, for a clean SC device, the power varies between 6–7 W, with an average measured power of 5.8 W over 2 minutes. However, for the inoculated FR4 and SC device, it is observed that the input power follows a steadily increasing trend, starting from ∼2 W and gradually increasing to the input power values noted in the case of the clean FR4 or SC devices.

### Temperature Measurements

A FLIR A320 ® Infrared camera was used to measure the substrate surface temperature during plasma generation. The infrared camera uses an uncooled micro-bolometer to detect infrared energy (heat) and converts it into an electronic signal, which is then processed to produce a thermal image that can be processed to obtain surface temperature.

In order to obtain the thermographic image of each plasma device, while it was being operated, the plasma device was powered for 2 minutes, during which thermographic images of the plasma device were obtained by the infrared camera at a sampling rate of 0.5 Hz. After turning off the plasma device, the camera continued to record images for another 2 minutes, thus yielding 48 frames. These images were transferred in real-time to a computer, wherein they were subjected to additional data processing.

**Figure 13:**
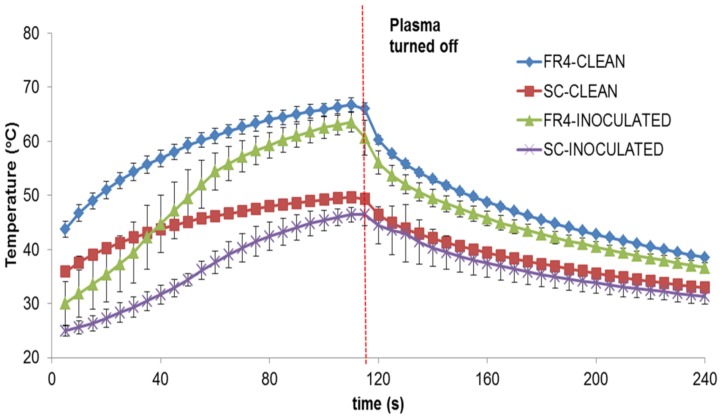
Comparison of surface temperatures during plasma generation for clean and inoculated FR4 and SC devices, measured using an infrared camera. Devices were powered for 2 minutes.

Figure 13 below shows the comparison of substrate temperature for FR4 and SC dielectrics run clean or inoculated with *E. coli*. In order to compare substrate temperatures, the average temperature over the entire surface area of the substrate was calculated for each frame. This is then plotted against time (s). The average of three sets of data has been plotted in Figure 13. Note that the camera starts recording 5s after the plasma is turned on.

In Figure 13 above, it is evident that during plasma generation the FR4 surface is at a much higher average temperature than the SC surface in both the clean and inoculated cases. However, average surface temperatures range between 30°C–66°C (for FR4) and 20°C–49°C (for SC). Ayan et. al. [20] evaluated the heating effect of DBD plasma, in which they measured surface temperatures of 310–350 K (36.8°C –76.8°C) and rotational temperatures (gas temperatures) of 340–360 K (66.8°C–86.8°C), using both sinusoidal and microsecond pulsed discharge. Their results indicated that in both types of discharge, while the rotational (gas) temperature was lower, the vibrational temperature was an order of magnitude higher than the rotational temperature, thus probably enhancing chemistry and leading to sterilization. Our measured surface temperatures are cooler than those of Ayan et. al. and thus are less likely to contribute to microbial killing, although temperatures of 56°C are sufficient to denature the 30S ribosomal subunit [21].

From Figure 13, it is also observed that while the standard deviation of the temperature measurements in the clean FR4 and SC cases is minimal, it is slightly higher in the case of temperature measurements in the inoculated cases. This standard deviation is greater during the first 60s for inoculated FR4, while for SC, it is greater during 60–105s. The significance of this will be discussed later on while discussing the effect of the liquid on the dielectric surface. It is to be noted that the slightly large variation in standard deviation for inoculated SC, during the latter part of the curve after plasma is turned off, stems from the fact that for one of the temperature data-sets, the plasma was turned off at 120±10s.

Thus, from the results above, it is observed that the sterilization plots (Figures 5, 6, 7) prove that the DBD surface plasma experimental setup can completely sterilize vegetative pathogens in 2–3 minutes, starting from an initial concentration of 10^7^ cfu. Plasma treatment using the setup can also lead to complete inactivation in bacterial spores in 20 minutes, starting from an initial concentration of 10^6^ cfu. Furthermore, spectroscopic, ozone, power and temperature studies show higher spectroscopic intensities, greater ozone levels, higher input power and higher surface temperatures in the case of the FR4 dielectric as compared to the semi-ceramic (SC) dielectric. It is also noted that these parameters differ widely between a clean and inoculated case for both dielectrics. The significance of these results is discussed in the next section.

## Discussion

The current paper examines sterilization using a DBD surface plasma generator in room air. Sterilization trials using different test pathogens were conducted. Killing times of 2 minutes or less were noted for vegetative cells (*E. coli* and *S. cerevisiae*). *G. stearothermophilus* spores required 20 minutes. These times are faster than previous reports involving volume plasma. Hence, we have been able to obtain fast sterilization using a simple experimental setup. Current efforts are being targeted at making this setup portable and scaling it up in size to sterilize larger surfaces.

The purpose of this paper was to study two factors affecting the process of plasma sterilization. One was to analyze the effect of different dielectric substrates on the process of plasma sterilization. Two dielectric substrates were used. One was standard FR4, while the other was a semi- ceramic laminate. The dielectric constant (k = 3.8–4.5 mean = 4.15) for the former is not a tightly controlled variable, while the latter has a dielectric constant of k = 3.00±0.05. Thus, the dielectric constant of FR4 is an average of 28% higher than the SC.

The dielectric constant of a material is the ratio of amount of electrical energy stored in a material by an applied voltage, relative to that stored in vacuum. Any of the devices described in this paper can be considered as a parallel plate capacitor, using a dielectric material of dielectric constant ‘k’. Then, the capacitance of such a system is
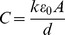
where ε_0_ = absolute permittivity of air, A = surface area of the top surface of the device &‘d’ = the thickness of the dielectric layer. The energy stored in a parallel plate capacitor is 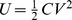.

Hence, for the devices considered in this paper, energy stored in the device is directly proportional to the capacitance of the system, which in turn is directly proportional to the dielectric constant of the material. Hence the FR4 device, which has a higher dielectric constant than the SC device, has more energy stored in the dielectric layer, which explains the results noted in Figure 6 i.e. complete sterilization is achieved faster (t = 90s) for FR4 as compared to semi-ceramic (SC) dielectric (t = 120s).

This difference is also supported by spectroscopic studies, which show that while plasma generated in both cases show similar spectra, the emitted intensities differ in both cases i.e. emitted intensity is higher by about 20–30% in the case of FR4. For the sake of simplicity, the spectral signatures for the semi-ceramic (SC) devices were not shown in this paper, as the peaks did not differ from those for FR4, just the intensity. Similarly, as shown in Figure 10 and 11, measured ozone levels show that the ozone levels produced in the case of FR4 devices (both clean and inoculated) are higher (25%–28%) than those produced in the case of semi-ceramic devices (both clean and inoculated). Additionally, Figure 13 shows that the maximum difference in average surface temperature during plasma generation between the FR4 and SC devices is ∼18°C. Such a slim difference in average surface temperature leads us to believe that temperature is not the differentiating factor between sterilization times for FR4 and SC. The FR4 and SC plasma devices, when compared, differ only in that they are constructed out of different dielectric materials. This would imply that the difference in sterilization times noted between FR4 and SC plasma devices is due to the difference in dielectric constants. Thus, FR4 proves to be a much more efficient dielectric surface for plasma sterilization.

**Figure 14:**
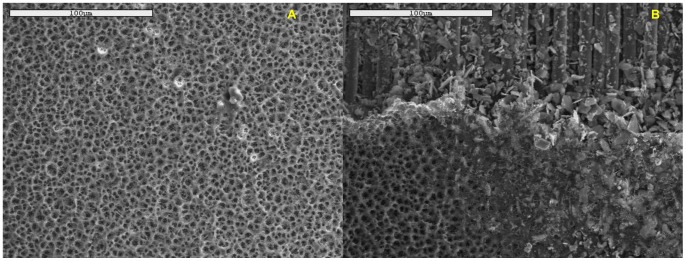
Preliminary SEM studies depicting the appearance of the dielectric substrate in (A) a fresh unused plasma device (B) a plasma device that has been powered continuously for 30 minutes. The devices shown have been imaged at a magnification of 500×. Comparing (A) and (B), it is evident that while (A) shows a fresh dielectric surface, (B) shows a degraded dielectric surface, wherein it appears that the top-layer seems to have eroded away, thus displaying the underlying fibers. A more detailed study of this degradation is ongoing and will be reported later.

However, this advantage is offset by the disadvantage posed by the faster degradation of the FR4 dielectric. Preliminary scanning electron microscopy (SEM) studies indicated that after about 30 minutes of plasma generation, the FR4 dielectric starts degrading. This is shown below in Figure 14. One way to explain this might be because FR4, which has a higher dielectric constant, requires a higher input power for plasma generation, which in turn leads to higher surface heating, thus leading to faster breakdown of the dielectric surface in the case of FR4. A more detailed investigation of the surface degradation of both FR4 and SC dielectrics is ongoing and will be reported later.

A note on the operation of DBD plasma devices and the high levels of ozone emission has to be made. During these experiments, the ozone levels within the laboratory did not rise to unsafe levels, but local ozone levels directly above the device did exceed safe levels(as seen in the enclosed chambers). Safe permissible levels for ozone are 0.1 ppm, as per the Occupational Safety and Health Administration (OSHA), 0.1 ppm, as per the National Institute of occupational safety and health (NIOSH) and 0.05 ppm, as per the Food and Drug Administration for indoor medical devices (FDA). To prevent unsafe ozone exposure, experiments were conducted in an acrylic enclosure that was vented away from the operator at the end of the experiment. Charcoal was used as an adsorbent either glued to a wire mesh cage placed over the test bench or in respirators.

The other factor analyzed in this study was the effect of having liquid present on the surface of the plasma device. The evaporation of the liquid *E. coli* sample deposited upon the device surface follows a pattern. Initially the bacterial sample deposited covers the entire electrode surface area, and plasma is visible only around the edges of the electrode. As time progresses, the sample begins to evaporate around the outer edges of the electrode. Gradually, this evaporation begins to spread to other parts of the electrode, until eventually plasma covers the entire electrode surface area. This usually occurs at around t = 90s for the FR4 dielectric and just before t = 120s for the semi-ceramic (SC) dielectric. A steep drop in pathogen concentration is also noted precisely at these time points (Figure 6).

Spectroscopic, ozone, power and temperature data uniformly show that plasma is repressed while visible liquid is present on the test devices. The spectral peaks (Figure 8, 9) are noted at the same wavelengths at each time point; however, their intensities increase as the liquid evaporates. Similarly, it is observed that as the liquid sample evaporates, rate of production of ozone increases (Figure 10, 11). Additionally, temperature data (Figure 13) demonstrates that the standard deviation of temperature measurements in inoculated cases is especially large during the first 60s (for FR4) and during 60–105s (for SC). Visibly, it is observed that the liquid starts evaporating rapidly during these exact time intervals for both dielectrics, which is why a large variation in surface temperature can be observed. Once the liquid is completely evaporated, during the last 30s and 5–10s for FR4 and SC respectively, very little standard deviation is observed.

**Figure 15:**
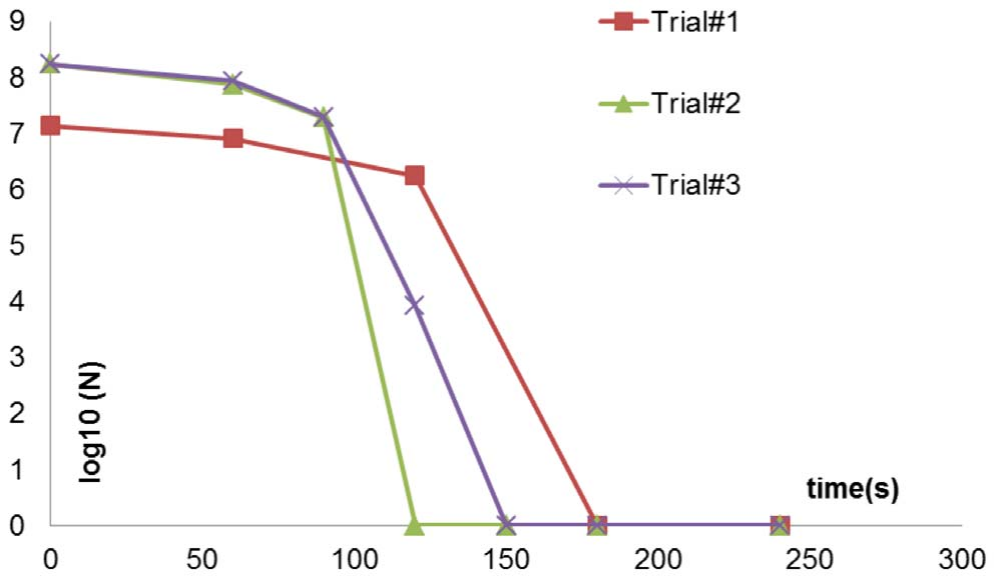
Sterilization plots for inoculation volume = 40 µl of *E. coli*.

When the same number of organisms was deposited in a 40 µL volume instead of the standard 20 µL inoculation volume, the “passive phase” wherein there is little or no loss of viability (Figure 6) was extended by about 30s here. Thus, the rapid drop in *E. coli* concentration occurs at t≥120s, as opposed to 90s in the case of the lower inoculation volume (20 µl). This is shown below in Figure 15.

Hence, the point at which all the liquid covering the electrode evaporates and plasma covers the entire electrode surface area is the point at which there is a rise in surface temperature, input power, emitted ozone levels and spectroscopic intensity. This is also the point where the steep drop in pathogen concentration occurs, thus indicating that there is a threshold time-point at which complete sterilization occurs. Hence in our case, liquid seems to inhibit plasma generation and killing and this should be taken into account when designing surface sterilization systems.

One way to explain this liquid dependence is in terms of capacitance. As per the ‘parallel-plate capacitor’ theory discussed earlier, if the FR4/SC plasma device is considered as a capacitor of capacitance (C_1_), then the liquid layer spread uniformly on top of the device can be considered as a second capacitor of capacitance (C_2_), connected in ‘series’ with C_1_. Thus the combined capacitance of this system would be
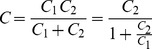



The impedance Z_1_ of the liquid layer varies inversely with the amount of liquid present on the surface of the device i.e. as the liquid evaporates, impedance Z_1_ decreases. Since Z_1_ is inversely proportional to capacitance, C_1_ (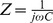), it follows that as Z_1_ decreases, C_1_ increases. Following this, as C_1_ increases, the capacitance of the overall system (C) increases and thus, the energy stored in the system (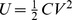) increases, proving that the amount of the liquid on the surface of the plasma device is actually detrimental to the performance of the plasma device as a sterilizer. This is mirrored in Figure 15 i.e. more the inoculation volume, more the amount of liquid covering the electrode surface, less the input power absorbed and hence more the sterilization time.

**Figure 16:**
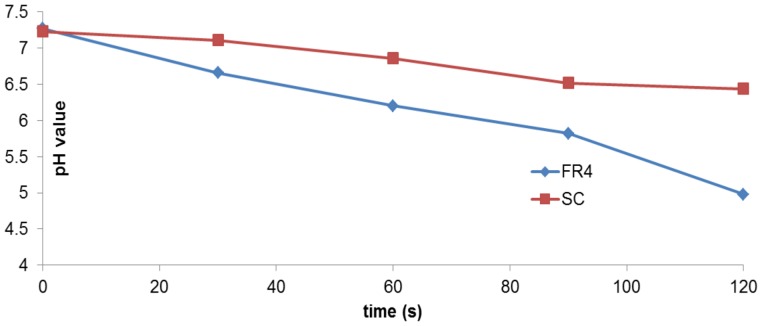
Plot of pH values, obtained by rinsing devices with Millipore water after plasma generation and measuring the pH value of this water in each case. Both FR4 and SC dielectrics are compared. pH values do not change as much in the case of SC, as compared to the case of FR4.

Oehmigen et.al. [22] reported experiments wherein they examined the role of acidification in influencing antimicrobial activity due to DBD plasma exposure. They concluded that plasma treatment of non-buffered liquids by indirect surface DBD resulted in acidification and thus, inactivation of suspended bacteria. When they tested the same theory with buffered solutions, they noted that pH decrease was avoided and therefore, antimicrobial plasma activity was reduced. It was suggested that reactive species from the plasma generation are the cause of liquid acidification and bactericidal activity. Along similar lines in our study, plasma devices inoculated with 20 µl of *E. coli* and plasma treated for Δt = 30,60,90,120s were placed in sterile bags and thoroughly rinsed with 1 ml of Type 1 (ultrapure) Milli-Q® water. For each sample, the pH of the corresponding volume of water was measured using an Accumet® AB 15 pH meter (accuracy of ± 0.01). The process was repeated for both FR4 and SC dielectric devices. Before measuring the pH, the meter was standardized using pH buffer solution. The pH of LB broth used to make the *E. coli* sample was measured as 7.16 and that of the *E. coli* sample itself was measured to be 6.77. The variation of pH is given below in Figure 16.

Figure 16 above indicates that the reduction of pH is greater in the case of FR4 as compared to SC. However unlike the drastic reduction in pH values noted by Oehmigen et.al. [22], there is not a strong pH change in our results (both FR4 and SC). Thus, it is most likely that acidification plays some role but not a major one in bacterial cell death. Note that the pH value does not vary much, except during the last 30s (for FR4) and not at all for SC. This is again indicative of the effect of the liquid on the dielectric surface. Since the liquid bacterial sample deposited on the dielectric substrate does not evaporate until the very end of the sterilization time interval (for both FR4 and SC), the pH does not change very much until the very end. This confirms that the liquid deposited on the dielectric substrate inhibits plasma generation and hampers the sterilization process.

In conclusion, this paper describes the usage of a DBD surface plasma generator using air as the working gas to implement sterilization. Complete sterilization, starting with an initial concentration of 10^6^–10^7^ cfu, is achieved within 90s to 120s (for vegetative pathogens) and within 20 minutes (for spores). FR4 is more efficient in this aspect, as compared to SC. The intensity of spectral peaks, amount of ozone generated, the absorbed input power and the surface temperature during plasma generation are all higher in the case of FR4. However, preliminary SEM studies also indicate a faster degradation of the FR4 dielectric. Thus, a trade-off may be required between faster sterilization times and durability of the plasma devices.

Spectroscopic studies show that the spectral pattern characteristic of the DBD plasma generated in this setup shows intensity peaks at wavelengths characteristic of the 2^nd^ positive system of N_2_. FR4 and SC plasma devices show intensity peaks at same wavelengths, although they differ in intensity values shown at each wavelength. Future studies will include investigating whether this difference in intensity value is integral to the difference in sterilization times when using FR4 and SC. Similarly, ozone studies show that a clean FR4 device produces more ozone than a clean semi-ceramic (SC) device. Additionally an inoculated FR4 or SC device produces less ozone than the respective clean devices. Temperature studies show that the surface temperatures reached during plasma generation are in the range of 30^°^C–66°C (for FR4) and 20°C–49°C (for SC). pH studies indicate a slight reduction in pH value due to plasma generation, which coupled with temperature studies implies that while temperature and acidification may play a role in DBD plasma sterilization, these are not the dominant roles.

Plasma generation and sterilization are also inhibited by liquid on the electrode, as evidenced by spectroscopic, ozone, temperature and absorbed power measurements in a clean case as compared to an inoculated case. Future studies will include the investigation of sterilization times needed when plasma devices are inoculated and allowed to dry. However, it is clear that the experimental setup will have to be designed, keeping this liquid dependence in mind. Thus, our work shows that DBD surface plasma generators hold great promise for rapid and economical sterilization.

## References

[1] Conrads H, Schmidt M (2000) Plasma generation and plasma sources. Plasma Sources Sci Technol 9: 441–454.

[2] Kogelschatz U (2003) Dielectric barrier Discharges: Their History, Discharge Physics and Industrial Applications. Plasma Chemistry and Plasma Processing 23(1): 1–46.

[3] Moisan M, Barbeau J, Moreau S, Pelletier J, Tabrizian M, et al (2001) Low-temperature sterilization using gas plasmas: a review of the experiments and an analysis of the inactivation mechanism. International Journal of Pharmaceutics 226(1): 1–21. 11532565 10.1016/s0378-5173(01)00752-9

[4] Laroussi M (2005) Low Temperature Plasma-based Sterilization: Overview and State- of- the-Art. Plasma Processes and Polymers 2(5): 391–400.

[5] Gallagher M, Vaze N, Gangoli S, Vasilets VN, Gutsol AF, et al (2007) Rapid inactivation of airborne bacteria using atmospheric pressure dielectric barrier grating discharge. Plasma Science, IEEE Transactions on 35(5): 1501–1510.

[6] Menashi WP (1968) U S Patent No. 3383163. Washington, DC: U S Patent and Trademark Office.

[7] Hury S, Vidal DR, Desor F, Pelletier J, Lagarde T (1998) A parametric study of the destruction efficiency of bacillus spores in low pressure oxygen-based plasmas. Lett Appl Microbiol 26(6): 417–421. 9717311 10.1046/j.1472-765x.1998.00365.x

[8] Lerouge S, Wertheimer MR, Marchand R, Tabrizian M, Yahia LH (1999) Effect of gas composition on spore mortality and etching during low-pressure plasma sterilization. J Biomed Mater Res 51(1): 128–135. 10.1002/(sici)1097-4636(200007)51:1<128::aid-jbm17>3.0.co;2-# 10813754

[9] Moreau S, Moisan M, Tabrizian M, Barbeau J, Pelletier J, et al (2000) Using the flowing afterglow of a plasma to inactivate B. subtilis spores: Influence of the operating conditions. J Appl Phy 88(2): 1166–1174.

[10] Ying J, Chunsheng R, Zhilong X, Dezhen W, Younian W, et al (2006) Comparison of yeast inactivation treated in He, Air and N2 DBD Plasma. Plasma Science and Technology 8(6): 720.

[11] Sladek REJ, Filoche SK, Sissons CH, Stoffels E (2007) Treatment of Streptococcus mutans biofilms with a nonthermal atmospheric plasma. Lett Appl Microbiol 45(3): 318–323. 17718846 10.1111/j.1472-765X.2007.02194.x

[12] Kalghatgi S, Kelly CM, Cerchar E, Torabi B, Alekseev O, et al (2011) Effects of non-thermal plasma on mammalian cells. PLoS One 6(1): e16270. 21283714 10.1371/journal.pone.0016270 PMC3025030

[13] Joshi SG, Cooper M, Yost A, Paff M, Ercan UK, et al (2011) Nonthermal dielectric barrier discharge plasma-induced inactivation involves oxidative DNA damage and membrane lipid peroxidation in Escherichia coli. Antimicrobial agents and chemotherapy 55(3): 1053–1062. 21199923 10.1128/AAC.01002-10 PMC3067084

[14] Lerouge S, Fozza AC, Wertheimer MR, Marchand R, Yahia LH (2000) Sterilization by low-pressure plasma: the role of vacuum-ultraviolet radiation. Plasmas and Polymers 5(1): 31–46.

[15] Laroussi M, Leipold F (2004) Evaluation of the roles of reactive species, heat and UV radiation in the inactivation of bacterial cells by air plasmas at atmospheric pressure. International Journal of Mass Spectrometry 233(1): 81–86.

[16] Dobrynin D, Fridman G, Friedman G, Fridman A (2009) Physical and biological mechanisms of direct plasma interaction with living tissue. New Journal of Physics 11(11): 115020.

[17] Yu H, Xiu ZL, Ren CS, Zhang JL, Wang DZ, et al (2005) Inactivation of yeast by dielectric barrier discharge (DBD) Plasma in helium at atmospheric pressure. Plasma Science, IEEE Transactions on 33(4): 1405–1409.

[18] Mastanaiah N, Wang CC, Johnson JA, Roy S (2011) A computational diagnostic tool for understanding plasma sterilization. In 49th AIAA Aerospace Sciences Meeting and Exhibit, AIAA Paper.

[19] Pearse R, Gaydon A (1976) The identification of molecular spectra. London: Chapman and Hall.

[20] Ayan H, Fridman G, Staack D, Gutsol AF, Vasilets VN, et al (2009) Heating effect of dielectric barrier discharges for direct medical treatment. Plasma Science, IEEE Transactions on 37(1): 113–120.

[21] Mackey BM, Miles CA, Parsons SE, Seymour DA (1991) Thermal denaturation of whole cells and cell components of Escherichia coli examined by differential scanning calorimetry. J Gen Microbiol 137(10): 2361–2374. 1722814 10.1099/00221287-137-10-2361

[22] Oehmigen K, Hahnel M, Brandenburg R, Wilke C, Weltmann KD, et al (2010) The role of acidification for antimicrobial activity of atmospheric pressure plasma in liquids. Plasma Processes and Polymers 7(3–4): 250–257.

